# Factors influencing menstrual hygiene knowledge, attitudes, and practices among adolescent girls in African rural schools: scoping review

**DOI:** 10.3389/frph.2025.1553101

**Published:** 2025-08-12

**Authors:** Mosotho Zenia Tshivule, Molatelo Melitah Rasweswe, Tebogo Maria Mothiba, Mamare Adelaide Bopape

**Affiliations:** ^1^ Department of Nursing Science, University of Limpopo, Polokwane, South Africa; ^2^ Research Office, University of Limpopo, Polokwane, South Africa; ^3^ Faculty of Health Science, University of Limpopo, Polokwane, South Africa

**Keywords:** menstrual hygiene, adolescent girls, rural schools, Africa, knowledge, attitudes, practices, factors

## Abstract

Menstrual hygiene management (MHM) is a critical component of adolescent health and well-being, particularly in rural African schools where cultural, economic, and infrastructural challenges persist. This scoping review followed the steps proposed by Arksey and O'Malley to explore the factors influencing menstrual hygiene knowledge, attitudes, and practices among adolescent girls in rural African settings. The review systematically analysed literature conducted in diverse African rural public schools. Findings reveal that many adolescent school girls lack comprehensive knowledge about menstruation, due to unreliable or insufficient sources of menstrual hygiene management information and unpreparedness for menarche. Furthermore, their menstrual hygiene practices are shaped by various sociocultural and religious influences. There is also resource limitation, related to availability and cost of menstrual hygiene management supplies, and water, sanitation, and hygiene (WASH) infrastructure in rural schools. These challenges have a significant impact on school attendance, academic performance, and the overall quality of life for adolescent girls. The review practice. It emphasizes the importance of multi-sectoral approaches in supporting adolescent girls in rural African schools and advocates for further research to address persistent knowledge and practice gaps. There is also a need for integrated interventions, including menstrual health education, improved WASH infrastructure, and the provision of affordable menstrual products. Addressing these factors holistically can enhance menstrual hygiene management, reduce stigma, and promote gender equality in education.

## 1 Introduction

Menstrual hygiene plays a crucial role in the overall well-being of adolescent girls by promoting their physical health, emotional well-being, and social development (1). However, it is often overlooked in public health agendas, especially in low- and middle-income countries (LMICs) (2). Menstrual hygiene management (MHM) encompasses the various methods and products employed by women and girls to regulate their menstrual cycles and address the associated challenges effectively. The subject encompasses the management of debris, the maintenance of personal hygiene, and the utilisation of menstrual products (3). In the interim, it is essential to recognise that menstrual health embodies a comprehensive state of complete physical, mental, and social well-being, rather than being merely characterised by the absence of disease or infirmity, especially within the framework of the menstrual cycle (4). MHM is globally influenced by multiple factors, including access to sanitation facilities, cultural taboos, socioeconomic status, and education (5). Cultural taboos surrounding menstruation are deeply ingrained, and girls often face shame and embarrassment during their menstrual periods (6). The socioeconomic disparities mean that many families cannot afford sanitary products, forcing girls to use unsafe alternatives (7). The lack of education on menstrual hygiene further exacerbates the problem, as many girls have little or no knowledge of how to manage their menstruation safely (8).

In response to these challenges, global and regional initiatives have been implemented to improve menstrual health. In South Asia, particularly in India and Nepal, governments have introduced large-scale programs such as India's Menstrual Hygiene Scheme, which provides subsidized sanitary pads and awareness campaigns to adolescent girls in rural areas (9). Nepal's National School Health and Nutrition Strategy integrates MHM education into the school curriculum and includes the construction of gender-friendly WASH facilities (10). Most countries in the United States and Brazil have initiated public policies to eliminate the “tampon tax” and incorporate menstrual education into national curricula, alongside efforts to distribute menstrual products in schools (11).

At the global level, the World Health Organization (WHO) and UNICEF have emphasized effective MHM as a key driver of gender equity and public health. They have jointly released the *WASH in Schools for Girls* framework, promoting access to clean water, private sanitation, and menstrual hygiene education (12). These initiatives align with the Sustainable Development Goals (SDGs), particularly SDGs 3, 4, 5, and 6, which stress the importance of ensuring all girls manage their menstruation hygienically and with dignity (13). Despite these global efforts, in Sub-Saharan Africa, the situation remains particularly acute. Adolescent girls in this region often face cultural taboos that stigmatize menstruation, leading to silence around the topic (14). The region suffers from inadequate WASH infrastructure, especially in schools, making menstrual management difficult (15). A large proportion of rural schools lack proper toilets, clean water, and private changing spaces (16). Consequently, many girls resort to using unhygienic materials such as rags, leaves, or newspapers, increasing their risk of infection (17). These conditions often result in school absenteeism and academic underperformance (18).

In South Africa, although national policies such as the Adolescent Sexual and Reproductive Health and Rights Framework Strategy (2014–2019) have aimed to improve menstrual health, disparities remain, especially in rural provinces like Limpopo and the Eastern Cape (46). Schools in these areas often lack private toilets, clean water, and safe disposal methods for menstrual products, while cultural stigma continues to limit open discussion and learning (6, 19). These conditions leave many adolescent girls unprepared to manage their menstruation safely and with dignity (20). Furthermore, teachers in rural schools often lack training and resources to provide comprehensive menstrual health education. Combined with poverty, limited access to healthcare, and deep-rooted cultural beliefs, these factors contribute to poor menstrual hygiene practices that have long-term implications for the health and education of adolescent girls (21). While progress has been noted in urban areas, a critical gap persists in rural schools, where poverty, stigma, and poor infrastructure intersect to limit menstrual hygiene management.

The persistent challenges faced by adolescent girls in rural schools across Africa highlight the need for targeted, context-specific interventions that address both the structural and cultural barriers to effective MHM. As researchers and policymakers continue to push for improved access to sanitary products and WASH infrastructure, it is essential to ensure that these efforts reach the most vulnerable populations, particularly girls in rural areas. Addressing the factors that influence MHM in rural schools is critical for improving health outcomes, promoting gender equality, and ensuring that all girls can succeed academically. Moreover, this review examined the factors influencing menstrual hygiene knowledge, attitudes, and practices among adolescent girls in rural African schools.

## 2 Design and methods

A scoping literature review method, as proposed by Arksey and O'Malley (22), was conducted. To ensure a structured approach, the following five steps were adhered to: (1) Identifying the research question or objective. (2) Identifying relevant literature by searching electronic databases (3) selecting the eligible literature using a set of criteria. (4) Charting the Data. (5) Data collating, summarizing, and reporting the results.

### 2.1 Research question

What are the key factors influencing menstrual hygiene knowledge, attitudes, and practices among adolescent girls in rural schools of Africa?

### 2.2 Identifying relevant literature

Journal articles were obtained by searching electronic databases through PubMed, Google Scholar, ScienceDirect, Scholar, and the African Index Medicus Database. The terms used for search were factors “AND” menstruation “OR” menstrual “AND” hygiene “AND” knowledge “OR” attitudes “OR” practices “AND” adolescents “OR” girls “AND” rural schools “AND” Africa.

### 2.3 Selecting the eligible literature

#### 2.3.1 Eligibility criteria

This scoping review included studies that employed a quantitative research approach focusing on menstrual hygiene knowledge, attitudes, and practices among adolescent girls attending rural schools in Africa. Qualitative, mixed-methods, grey literature, opinion papers, clinical reports, and any literature review were excluded. Only quantitative studies were included to ensure the extraction of measurable data that could be compared across settings. Qualitative and mixed-methods studies were excluded to maintain methodological consistency and focus on empirical, quantifiable data. The search strategy was limited to papers published in the English language between 2014 and 2024, aligning with the review's focus on recent and relevant evidence within the last decade. To identify relevant studies, a comprehensive electronic search was conducted across multiple databases, including EBSCOhost, JSTOR, ScienceDirect, SAGE, SpringerLink, PubMed, Sabinet, and Google Scholar. The search strategy was restricted to publications with a geographic focus on African countries. Following the search, each member of the research team independently screened the titles and abstracts using Rayyan software to ensure objectivity and minimize selection bias. Articles that met the inclusion criteria were then subjected to a full-text review. Studies were excluded at this stage if they did not meet the eligibility criteria or if insufficient information was available to assess their relevance to the research question. Additionally, if a full-text article could not be accessed despite reasonable efforts, it was excluded, as it was not possible to confirm whether it met all the inclusion criteria.

### 2.4 Charting the data

After identifying and selecting relevant papers in the initial screening process, full-text articles were assigned to individual authors for reading and data extraction. Detailed information about resources was charted in a spreadsheet. Data extraction included the following items: author, year, and country; aim or objectives; design and data collection method; sampling and population; and key findings. Study records were tagged by the research question(s) that they addressed.

### 2.5 Data collating, summarizing, and reporting the results

**Figure 1:**
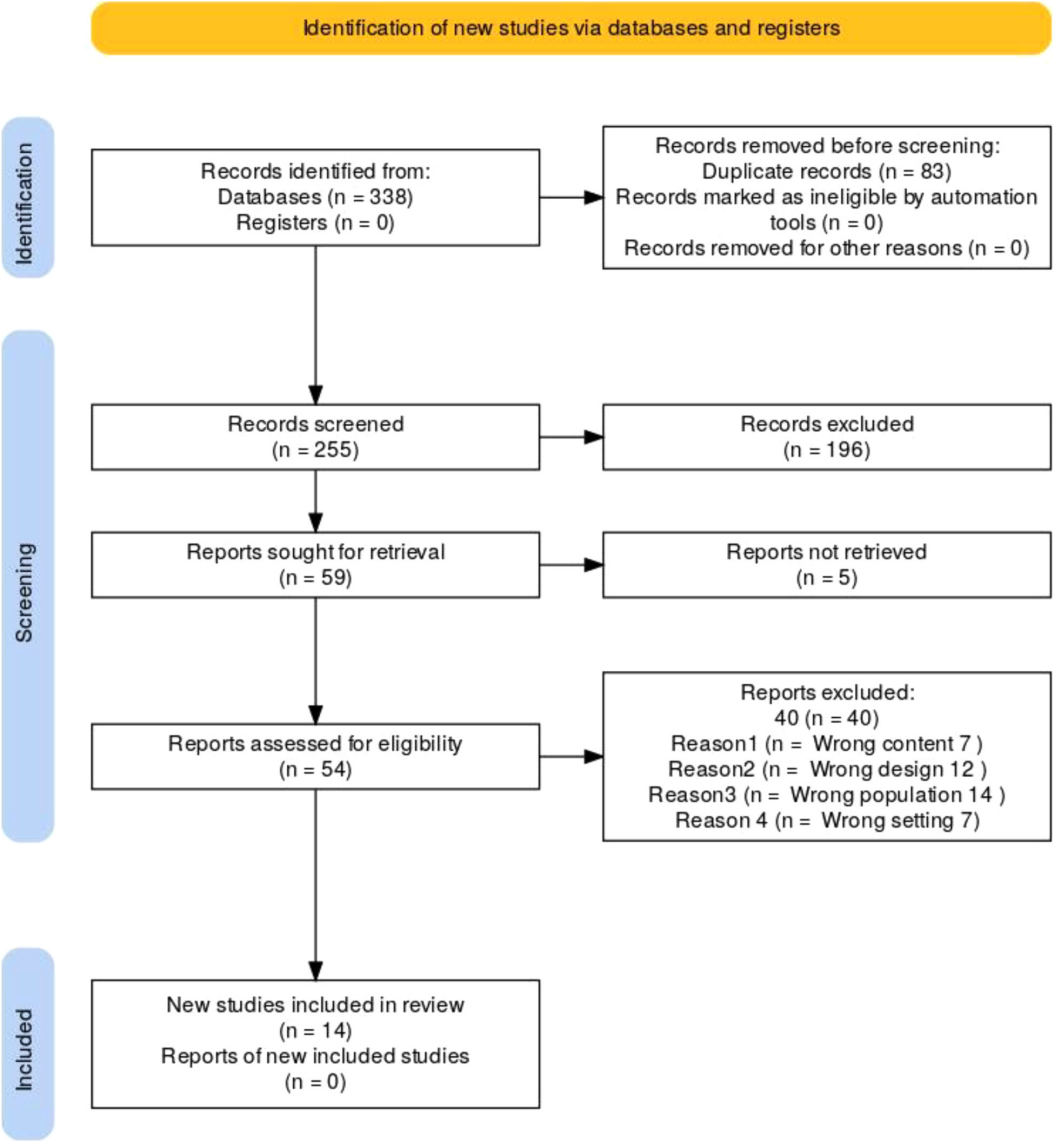
PRISMA flow diagram of search (adapted from 2020 PRISMA flow diagram).

A total of 14 articles were deemed eligible and included in the review (refer to Figure 1; Table 1). The data synthesis process involved a systematic approach to gathering, organizing, and summarizing information from scientific literature. This review aimed to comprehensively analyse factors that influence menstrual hygiene knowledge, attitudes, and practices among adolescent girls in rural African schools. Thematic analysis was employed to identify recurring themes. To synthesize the data comprehensively, related findings from the reviewed literature were systematically clustered to identify patterns and commonalities. These grouped findings were then analysed by examining the varied approaches adopted by different authors. Through an iterative process, the data were methodically coded and categorized, allowing for a refined understanding of the themes and trends emerging across the studies. Emerging patterns and concepts were refined through collaborative discussions among researchers, ensuring the themes accurately reflected the findings across the reviewed studies. Three themes and six subthemes emerged as discussed below.

**Table 1:** Studies included in the review (created by Tshivule MZ).

No	Author, year, and country	Aim or objectives	Design and data collection method	Sampling and population	Key findings	Quality appraisal (scale: h = high; l = low; nr = not reported)
1.	Onubogu et al. (2024) (1) Rural Anambra State, Nigeria	To examine the menstrual hygiene practices of adolescent girls in rural Anambra communities	Quantitative, Cross-sectional, and descriptive Self-administered semi-structured questionnaire	Multistage Stratified Random Sampling Public secondary school adolescent girls	Inadequate knowledge about menstrual hygiene, lack of facilities for changing, and unhygienic products among girls The need for active MHM teaching in primary school curriculum and school health programmes	(h) Aim and objectives clearly stated (h) The study design was clearly stated (h) Appropriate research methods
2.	Bulto (2021) (47) Oromia region, central Ethiopia	To determine the practice of MHM and associated factors in central Ethiopia	Quantitative, descriptive cross-sectional study Self-administered questionnaire	Systematic random sampling Adolescent girls from three schools in Holeta town	. Poor menstruation awareness and hygiene practices increase the risk of reproductive and genitourinary infections and cervical cancer	(h) Aim and objectives clearly stated (h) The study design is clearly stated (h) Appropriate research methods
3.	Belayneh and Mekuriaw (2019) (5) Gedeo zone Southern Ethiopia	To assess the Knowledge and menstrual hygiene practices of adolescent schoolgirls in Gedeo zone	Quantitative, cross-sectional Structured and interviewer-administered questionnaire	Multi-stage sampling Adolescent school girls.	Insufficient knowledge regarding menstruation and suboptimal menstrual hygiene practices	(h) Aim and objectives clearly stated (h) The study design was clearly stated (h) Appropriate research methods
4.	Ramathuba (2015) (6) Thulamela municipality Limpopo Province, South Africa	To assess the knowledge and practices of secondary school girls towards menstruation in the Thulamela municipality, Vhembe District, Limpopo	Quantitative, explorative descriptive Self-administered questionnaire	Convenience sampling Secondary school girls	Only 48% of adolescents prior to menarche possessed knowledge of hygienic menstrual practices	(h) Aim and objectives clearly stated (h) The study design clearly stated (h) Appropriate research methods
5.	Shumie and Mengie, (2022) (23) Mekdela district South Wollo zone Northeast Ethiopia	To identify knowledge and practice of menstrual hygiene, and associated factors	Quantitative, cross-sectional Structured self-administered questionnaire	The stratified sampling technique was used Female students from grades 9th to 12th in Mekidela secondary schools	Good knowledge and good practice of menstrual hygiene management, informed about menstruation before menarche, mainly from their mothers However, menstrual hygiene practice was unsatisfactory	(h) Aim and objectives clearly stated (h) The study design clearly stated (h) Appropriate research methods
6.	Kumbeni et al. (2020) (24) Rural northern Ghana	This study sought to investigate menstrual hygiene management among adolescent girls in junior high schools in rural northern Ghana	Quantitative, school-based cross-sectional study design Structured questionnaire	Multistage sampling technique School girls	The prevalence of good menstrual hygiene was 61.4%, despite inadequate sanitation facilities. The education of mothers and parents’ socio-economic status greatly influences menstrual hygiene management. use was linked to school attendance	(h) Aim and objectives clearly stated (L) The study design was clearly stated (h) Appropriate research methods
7.	Daniel et al. (2023) (17) Gimbi town, west Wollega zone. Western Ethiopia	To assess menstrual hygiene management practice and its associated factors among in-school adolescent girls in the secondary schools of Gimbi town, western Ethiopia	Quantitative, cross-sectional. Self-administered questionnaire and observational checklist	Stratified random sampling techniques. Adolescent girls in Gimbi town secondary schools	Menstrual hygiene management practice is poor, and they can't afford sanitary pads The girls absent themselves from school during their monthly periods	(h) Aim and objectives clearly stated (h) The study design is clearly stated (h) Appropriate research methods
8.	Hussein et al. (2022) (16) East Hararghe, Ethiopia	To assess practices of menstrual hygiene management and associated determinants among secondary school girls in East Hararghe	Quantitative cross-sectional survey. Self-administered questionnaire	Simple random sampling School girls	A high number of girls demonstrate a commendable understanding of menstrual hygiene; however, only two-thirds engage in effective menstrual hygiene management practices	(h) Aim and objectives clearly stated (h) The study design is clearly stated (h) Appropriate research methods
9.	Upashe et al. (2015) (25) Nekemte, Oromia, Western Ethiopia	To assess the knowledge and practice of menstrual hygiene among high school girls in Nekemte town	Quantitative cross-sectional Structured questionnaire	A multi-stage sampling Female high school students	Lack of knowledge and practice regarding menstrual hygiene, and few had good practices on menstrual hygiene	(h) Aim and objectives clearly stated (h) The study design was clearly stated (h) Appropriate research methods
10.	Gedefaw et al. (2019) (8) North Wollo Zone, Ethiopia	Assess knowledge, attitude, practice, and their associated factors of menstrual hygiene among high school students in North Wollo Zone, Woldia, Ethiopia	Quantitative institution-based cross-sectional study Structured, pretested questionnaire	Systematic random sampling technique Female high school students	Participants had high knowledge about MHM but low attitudes and practices related to it	h) Aim and objectives clearly stated (h) The study design is clearly stated (h) Appropriate research methods
11.	Girma et al. (2024) (26) Eastern Ethiopia	To assess MHM practice and associated factors among secondary school girls in eastern Ethiopia	Quantitative Institutional-based cross-sectional study design Self-administered structured questionnaire	Stratified sampling technique Secondary schoolgirls	Good menstrual hygiene. MHM practice was associated to school WASH features such as continuous water supply, MHM education, toilets, closed female restrooms, and private spaces	(h) Aim and objectives clearly stated. (h) The study design is clearly stated (h) Appropriate research methods
12.	Aluko et al. (2014) (19) Ile Ife, south-western Nigeria	assessed the knowledge and menstrual hygiene management (MHM) practices among in-school adolescents in Nigeria	Quantitative, descriptive, cross-sectional study Validated questionnaire and observational checklist	Multistage technique Female respondents between the ages 10 and 19	Mothers’ educational status influences knowledge on MHM. There is a good knowledge of MHM, but poor practice is observed. Poor sanitation and hygiene facilities in schools hinder safe MHM	(h) Aim and objectives clearly stated (h) The study design is clearly stated (h) Appropriate research methods
13.	Gultie et al. (2014) (20) Amhara Ethiopia	The objective of this study, therefore, is to assess the age of menarche and knowledge of adolescents about menstrual hygiene management in Amhara province	Quantitative, school-based, cross-sectional study Pretested and structured questionnaire	Multistage sampling technique Female students	The primary source of information about menstrual hygiene were teachers, followed by mothers. Lack of latrine and water supply seriously affects menstrual hygiene management and jeopardizes the physical and psychological health of school adolescents	(h) Aim and objectives clearly stated (h) The study design is clearly stated (h) Appropriate research methods
14.	Gorah et al. (2020) (27) Bokkos Local Government Area of Plateau State, Nigeria	The study was carried out to help find the evaluation of the knowledge, attitudes, and practices of female students towards menstrual hygiene management in the Bokkos Local Government area of Plateau state	Quantitative, survey research design A structured questionnaire	Multistage Female students	The participants are embarrassed by menarche. They have negative attitudes and practices towards menstrual hygiene. They use cotton wool, throwing menstrual waste in a dustbin	(h) Aim and objectives clearly stated (L) The study design is clearly stated (h) Appropriate research methods

#### 2.5.1 Theme 1: knowledge about menstrual hygiene in African rural schools

The literature review reveals that adolescent girls attending public rural schools in Africa frequently demonstrate limited knowledge of MHM (5) contributing to difficulties in fostering positive attitudes and practicing effective hygiene (1). This lack of understanding exacerbates challenges in menstrual management. Key aspects explored include inadequate knowledge about menstruation, unreliable or insufficient sources of MHM information, and unpreparedness for menarche, all of which highlight the need for targeted interventions to address these knowledge gaps and promote comprehensive menstrual education.

#### 2.5.2 Subtheme 1.1: inadequate knowledge about menstruation

The studies revealed that adolescent girls in rural African schools often lack adequate knowledge about menstruation (47). This illustrates that menstrual health and hygiene (MHH) pose considerable public health challenges in rural Africa, where MHH is frequently underrecognized and insufficiently understood (8). The concept of menstrual health and hygiene (MHH) incorporates not only the physical and psychological aspects of menstruation but also the broader systemic factors that link menstruation to health, wellbeing, gender equality, education, equity, empowerment, and human rights (28). The insufficient understanding of menstrual hygiene management within rural African schools significantly impedes girls' capacity to navigate menstruation with confidence. A study conducted in rural Ethiopia highlights that a lack of adequate knowledge regarding menstrual health and hygiene (MHH) significantly impedes girls’ capacity to manage menstruation with confidence, thereby exacerbating stigma and contributing to absenteeism in educational settings (26).

Numerous adolescent girls in underprivileged public schools possess insufficient knowledge regarding menstruation, resulting in negative attitudes and inadequate hygiene practices. In contrast, a study conducted at Mekidela secondary schools, Ethiopia reported good knowledge and practice of menstrual hygiene management amongst girls in grades 11 and 12 (23). The onset of puberty frequently aligns with this knowledge gap, which is intensified by societal discomfort and misinformation among adults, including parents and educators, who refrain from discussing sexual and reproductive health topics at an early age (6). While policies such as South Africa's Comprehensive Sexuality Education (CSE) framework and Kenya's Free Sanitary Pads Initiative aim to address menstrual health gaps, their implementation in rural schools remains inconsistent. For instance, studies in Kenya found that while the provision of free sanitary pads improved school attendance, the lack of complementary menstrual education limited the initiative's broader impact (48, 49).

These challenges are exacerbated by limited access to resources such as sanitary products and proper sanitation facilities in rural schools (19). The deficit hampers their ability to manage menstruation effectively, influencing their physical, mental, and social well-being. Therefore, targeted education and open dialogue are essential for enhancing menstrual health outcomes for girls in rural African communities. Strengthening policy implementation with targeted monitoring mechanisms is essential to ensure rural schools can deliver both menstrual products and comprehensive education. Additionally, comprehensive MHM education programs that integrate accurate knowledge, supportive school environments, and community engagement are essential to address these gaps effectively.

#### 2.5.3 Subtheme 1.2: unreliable sources of knowledge for menstrual hygiene management

The management of menstrual hygiene represents a significant factor in the health and well-being of women and girls. Access to accurate and reliable information regarding menstrual health management remains a considerable challenge in numerous regions globally, with a particular emphasis on Africa. The absence of adequate information may lead to adverse health outcomes, including infections, discomfort, and social stigma (23). In numerous rural African educational institutions and communities, there is a notable absence of comprehensive sex education, particularly regarding menstruation and menstrual hygiene management (MHM). In numerous rural schools across Africa, adolescent girls encounter inconsistent and frequently unreliable sources of information regarding menstruation (26). Parents, especially mothers, serve as fundamental sources of information; however, they may experience a deficiency in confidence or possess incomplete knowledge, which can hinder their ability to guide their daughters (6) effectively. Educators, an essential resource, their educational provision (17). Information derived from peer sources, although readily available, often suffers from incompleteness or is marred by inaccuracies, thereby intensifying existing misconceptions (29). In Ethiopia and Malawi, adolescent girls indicated a dependence on peers or mothers for menstrual information, which frequently proved to be incomplete or inaccurate, thereby perpetuating stigma and inadequate menstrual management practices (30, 31). The identified challenges underscore the necessity for the implementation of standardised and culturally relevant menstrual health and hygiene education within rural educational institutions.

There is also a scarcity of healthcare providers, particularly those equipped to provide accurate MHM information to adolescent girls in schools. This limitation often stems from factors such as geographical remoteness, inadequate infrastructure, and a shortage of trained medical personnel (24). As a result, many girls rely on unreliable sources of information, including cultural myths and taboos, which can lead to harmful practices and negative health consequences. These traditional beliefs, while rooted in cultural heritage, may be outdated and no longer aligned with current scientific understanding. The language barrier further exacerbates the challenge of accessing accurate MHM information in rural African regions (21). In many rural schools, information materials and educational programs are primarily available in English or other official languages, which may not be understood by adolescent girls who primarily speak local languages.

The scarcity of healthcare providers in many African rural schools, coupled with language barriers, significantly hinders adolescent girls' access to accurate and up-to-date information about menstrual hygiene. The lack of access to accurate information has significant implications for girls' health and well-being. For example, unsanitary materials or improper hygiene practices can lead to infections such as urinary tract infections and reproductive tract infections (19). Furthermore, a lack of knowledge about menstrual cycles and hygiene management can lead to unnecessary suffering. Furthermore, misinformation and taboos can contribute to social isolation and discrimination, negatively impacting mental health (n Additionally, girls who lack access to sanitary products or information may miss school during their periods, affecting their education and future opportunities.

Addressing the problem of unreliable and insufficient sources of MHM information amongst adolescent girls in schools requires a multi-faceted approach. Schools and communities should provide age-appropriate, comprehensive sex education that includes information about menstruation, MHM, and reproductive health. Target schools to help dispel myths and promote accurate information. By addressing the root causes of unreliable and insufficient information about MHM in schools, we can improve the health and well-being of adolescent girls in Africa.

#### 2.5.4 Subtheme 1.3: unpreparedness for menarche

Menarche is a significant milestone in adolescence, which often arrives without sufficient preparation for many girls in rural settings. Research shows that many girls in African rural schools receive limited or no formal education about menstruation before menarche (16), leaving them unprepared for the physical and emotional changes and challenges associated with menstruation they experience. For instance, studies in Kenya and Uganda found that most girls were uninformed about menstruation until their first period, primarily due to the lack of comprehensive school-based menstrual education programs (32, 33). Research conducted in Nigeria indicates that 64% of girls in the Southeast and 77% in the Northwest areas were insufficiently educated about menstruation (34). In Uganda and Ethiopia, substantial obstacles to menstrual health and hygiene management (MHHM) were observed, with 68.3% of teenage girls in Ethiopia lacking sufficient information prior to onset and 60.3% unable to maintain good menstrual hygiene (17). The lack of comprehensive menstrual hygiene education in schools can have far-reaching consequences for adolescent girls' health and well-being. Research conducted in South Africa and Kenya has demonstrated that menstrual health topics are inconsistently integrated into school curricula, with many teachers lacking confidence or training to address these topics (31, 35). As a result, girls enter menarche unprepared, leading to poor menstrual management practices and heightened anxiety. Therefore, preparing girls for menarche requires proactive education, access to menstrual products, and supportive environments that normalize menstruation as a natural aspect of life.

This can lead to feelings of shame, anxiety, and adverse feelings toward menstruation, as well as isolation during menstruation and negative health outcomes such as infections and missed school days, which perpetuate stigma and hinder effective management. Furthermore, the lack of menstrual hygiene education can perpetuate harmful cultural beliefs and practices surrounding menstruation. In many African cultures, menstruation is often stigmatized and considered taboo, leading to restrictions on girls' activities and social interactions during their periods. These cultural norms can further exacerbate the negative impact of inadequate menstrual hygiene education, as girls may be reluctant to seek information or support from their families or communities. These results highlight the pressing need for enhanced educational and awareness programs centered on menstrual health.

Addressing the lack of menstrual hygiene education in schools is crucial for improving the health and well-being of adolescent girls in rural Africa. By providing comprehensive and age-appropriate menarche information, schools can empower girls to manage their menstrual cycles with confidence and dignity. By investing in menstrual health education and hygiene programs prior to menarche, we can help to break down the stigma surrounding menstruation and create a more equitable and inclusive learning environment for all girls attending rural schools.

#### 2.5.5 Theme 2: menstrual hygiene practices

The results showed that menstrual hygiene practices in African rural schools is a multifaceted issue shaped by different dynamics. The dynamics were related to sociocultural, religious, and individual factors, which directly influence the health and educational experiences of adolescent girls. Understanding the dynamics of menstrual hygiene practices requires examining how traditional beliefs and practices, religious doctrines, and societal norms influence menstrual hygiene behaviours.

#### 2.5.6 Subtheme 2.1: sociocultural and religious influences on menstrual hygiene practices

Sociocultural and religious beliefs play a significant role in shaping MHM globally. Moreover, in many African rural schools, sociocultural and religious factors significantly shape adolescent girls' menstrual hygiene practices. These beliefs can both positively and negatively influence the adolescent girls' experiences and behaviours. On one hand, some cultural practices, such as the use of traditional cloth pads, can be seen as environmentally friendly alternatives to commercial sanitary products (6). Furthermore, certain religious beliefs may encourage cleanliness and hygiene during menstruation, resulting in beneficial practices (5). Cultural taboos surrounding menstruation contribute to the perpetuation of misinformation and silence, thereby limiting adolescent girls' access to accurate knowledge. In Africa, numerous cultural and religious beliefs regarding menstruation are frequently linked to stigma, shame, and limitations on girls' activities and social interactions. Societal norms and cultural taboos often restrict open discussions about menstruation, perpetuating misinformation (36). The beliefs and taboos surrounding menstruation have a severe impact on girls' self-esteem and attitudes, perpetuating a cycle of poor MHM practices (24). Menstruation is frequently viewed via a cultural lens as unclean and impure, leading to social isolation. Girls were forbidden from participating in worship, cooking, or performing some domestic tasks. In India, women face restrictions on their daily activities, including sleeping during the day, bathing, wearing flowers, engaging in sexual interactions, physical contact with people, speaking loudly, and handling pickles (37). Shame, dread, worry, and depression were among the most triggered emotions (38). These issues are exacerbated by rural schools' inadequate access to essential resources, such as sanitary goods and proper sanitation facilities (39).

These negative perceptions can lead to harmful practices, such as the use of unhygienic materials, avoidance of school during menstruation, and limited access to menstrual hygiene education. Furthermore, sociocultural factors such as poverty and lack of access to sanitation facilities can exacerbate the challenges faced by adolescent girls (29). In many rural areas, girls may not have access to clean water, soap, or private toilets, making it challenging to maintain good menstrual hygiene. Additionally, economic constraints may limit their access to affordable sanitary products, forcing them to resort to less hygienic alternatives. These factors can contribute to increased risk of infections, discomfort, and absenteeism from school. This increases the risk of reproductive tract infections, which are prevalent in resource-limited settings (26, 52). Psychologically, inadequate knowledge fosters fear and anxiety around menstruation, contributing to mental health challenges like depression and reduced self-esteem (32). Socially, menstruation-related stigma isolates girls from peers and community activities, reinforcing gender inequalities and undermining social cohesion.

Religious beliefs also play a central role in shaping how menstruation is perceived and managed. In some communities, religious doctrines around purity and ritual cleanliness heavily influence the menstrual experience, with religious taboos restricting menstruating girls' participation in communal activities, such as attending church or school (6). For example, in some Christian and Islamic communities, menstruating girls are often expected to refrain from religious duties like praying or fasting, which can affect their mental health and self (40).

Individual factors, including knowledge, attitudes, socioeconomic status, and personal hygiene habits, significantly influence menstrual hygiene practices among adolescent girls in rural African schools. These factors are often intertwined with cultural and religious beliefs, which dictate how girls approach menstruation and manage their menstrual health. A lack of knowledge about menstrual hygiene is one of the most prominent individual factors influencing menstrual health practices in rural areas. Many girls in rural schools in Africa have a limited understanding of the biological process of menstruation and the necessary hygiene practices. According to a study in Uganda, many adolescent girls reported feeling ashamed and confused when they first menstruated because they had not been adequately prepared (33). This knowledge gap is often due to the insufficient integration of menstrual health education into school curricula, despite global frameworks like SDG 4 (Quality Education) emphasizing the importance of comprehensive health education (53).

Attitudes towards menstruation, shaped by cultural taboos and religious beliefs, affect how girls manage menstruation. A study in rural Kenya found that menstruation is often seen as a taboo subject, leading girls to hide their menstrual status from peers and teachers, which can affect their ability to practice good menstrual hygiene (36). Negative attitudes, such as the belief that menstruation is dirty or shameful, contribute to poor hygiene practices and result in lower self-esteem and academic performance (5). These attitudes often stem from cultural teachings and are exacerbated by inadequate menstrual health education and limited discussions in schools.

Socioeconomic factors, including access to sanitary products, also significantly influence menstrual hygiene practices. In rural areas, girls from lower-income families often lack access to affordable sanitary pads, leading them to resort to unhygienic alternatives, such as rags, cloth, or even leaves (41). A study in South Africa reported that 50% of adolescent girls in rural areas could not afford sanitary pads, which negatively impacted their menstrual hygiene practices and contributed to school absenteeism (18). The financial constraints of rural families, compounded by poor infrastructure, mean that menstrual health remains an unaddressed issue despite various policy frameworks, such as the African Union's Agenda 2063 and SDG 3 (Good Health and Well-being), calling for improved access to healthcare and hygiene products (50). Low socioeconomic status limits access to menstrual products, forcing reliance on unsafe alternatives. Research from Western Ethiopia highlights that girls with educated mothers are more likely to use safe materials, showing the impact of parental education (17). This ties to SDG 3 (good health and well-being) by underscoring equitable access to resources.

To address the impact of sociocultural and religious influences on menstrual hygiene practices, it is essential to promote open dialogue and education about menstruation. This aligns with SDG 5 (gender equality), which calls for addressing harmful practices and promoting empowerment. By challenging harmful stereotypes and misconceptions, we can empower girls to make informed decisions about their menstrual health. Additionally, providing access to affordable and sustainable menstrual products, as well as improving sanitation facilities in schools and communities, can significantly improve menstrual hygiene practices among adolescent girls. By addressing these issues, we can create a more supportive and inclusive environment for girls to manage their menstrual cycles with dignity and confidence.

#### 2.5.7 Theme 3: resource limitation and its impact on menstrual hygiene management

Resource limitations, particularly in rural African schools, critically affect MHM and have far-reaching implications for adolescent girls' education, health, and dignity. These limitations include restricted availability and affordability of menstrual hygiene products, inadequate water, sanitation, and hygiene (WASH) facilities, and a lack of comprehensive education about menstrual health.

#### 2.5.8 Subtheme 3.1: availability and cost of menstrual hygiene management supplies

In many rural African communities, the high cost of menstrual products such as sanitary pads makes them inaccessible to a significant portion of girls. For instance, studies in Kenya and Uganda have highlighted that some girls who cannot afford menstrual products resort to using unsafe alternatives like cloth or even leaves, increasing their risk of infections (32, 33).

The high cost of menstrual hygiene products, coupled with low household incomes, forces many girls to make difficult choices. When faced with financial constraints, they may opt for cheaper, less hygienic alternatives, such as rags or leaves. This can lead to health risks, including infections and discomfort, which can further disrupt their education (47). Girls who experience heavy menstrual flow or who cannot afford adequate protection may stay home during their periods to avoid embarrassment or discomfort. Thus, the unavailability of quality menstrual hygiene materials can also lead to absenteeism from school or reduce their school attendance due to embarrassment and discomfort during menstruation. Missing school during menstrual periods hinders adolescent girls' academic progress and future opportunities. This can have a significant impact on their academic performance and overall educational attainment (42). In addition to the direct costs of menstrual products, indirect costs, such as transportation to access healthcare facilities or purchasing sanitary pads, can further burden families. These economic barriers can disproportionately affect girls from low-income households, exacerbating existing inequalities and limiting their educational opportunities. This cycle of missed education can eventually perpetuate poverty and limit their potential. National efforts, such as the Kenyan government's provision of free sanitary pads to schoolgirls since 2011, have made some progress, yet disparities persist due to inconsistent supply chains and funding constraints (48, 49). Policies like South Africa's Zero VAT on sanitary products also attempt to alleviate cost barriers but require broader implementation across the continent to be impactful. Additionally, environmentally sustainable pads, such as those made from banana fibre, provide a feasible and economical alternative to disposable sanitary products, thereby enhancing affordability (43).

#### 2.5.9 Subtheme 3.2: WASH infrastructure in rural schools

The lack of gender-sensitive WASH facilities in schools remains a significant challenge, significantly impacting adolescent girls' menstrual hygiene and overall well-being. Gender-sensitive WASH refers to facilities that consider the specific needs and vulnerabilities of girls, particularly during menstruation (32). This includes providing private, clean, and functional toilets with adequate water supply, soap, and menstrual hygiene products. Studies have shown that inadequate WASH facilities can lead to increased absenteeism, poor academic performance, and adverse health outcomes for girls (26). However, many rural schools in Africa lack these basic amenities, forcing girls to make difficult choices that compromise their health and education. Only 47% of schools in sub-Saharan Africa have access to adequate sanitation facilities, and fewer offer private, safe spaces for girls to manage menstruation effectively (51).

The lack of private, clean, and functional toilets in schools poses a significant barrier to effective menstrual hygiene management. Moreover, the absence of privacy and dignity in toilet facilities can discourage girls from changing menstrual products, leading to discomfort and embarrassment. A study conducted by Sommer et al. (15) in Kenya found that the lack of adequate sanitation facilities, including separate toilets for boys and girls, was a major barrier to girls' education. Many girls reported feeling uncomfortable and embarrassed using shared toilets, particularly during their menstrual period. This can lead to absenteeism, as girls may avoid school during their menstrual cycle to avoid the discomfort and stigma associated with inadequate sanitation. Furthermore, the absence of clean water and soap can hinder proper menstrual hygiene practices. Without adequate water for washing, girls may be unable to clean themselves properly, increasing the risk of infections. This can lead to discomfort, pain, and potential health complications, further impacting their ability to concentrate in class.

Furthermore, the lack of clean water and adequate sanitation can make it difficult for girls to maintain proper hygiene during their periods. Without access to clean water for washing, girls may be forced to use unclean water sources, increasing their risk of infection. Additionally, the absence of soap and other hygiene products can hinder their ability to maintain personal hygiene. The lack of private, clean, and functional toilets in schools can have a profound impact on girls' education and overall well-being (23).

On the other hand, resource-strapped schools in rural Africa often face significant challenges in maintaining and improving their facilities, including WASH infrastructure (26). Limited funding hinders the construction, repair, and upkeep of toilets, water supply systems, and other essential amenities. This lack of investment led to dilapidated facilities, inadequate water supply, and poor sanitation conditions, directly affecting the health and well-being of students, particularly adolescent girls. Studies have shown that schools with inadequate facilities are more likely to experience higher rates of absenteeism, particularly among girls (29). When toilets are dirty, overcrowded, or lacking privacy, girls may avoid using them, especially during their menstrual periods.

To address this issue, it is crucial to prioritize investments in school infrastructure and allocate sufficient funding for the construction, maintenance, and repair of WASH facilities. By providing adequate resources, schools can ensure that girls have access to clean, safe, and private toilets, as well as clean water for handwashing and menstrual hygiene. This will not only improve the overall learning environment but also contribute to better health outcomes for adolescent girls in rural African schools. The Sustainable Development Goal (SDG) 6.2 emphasizes access to equitable sanitation and hygiene for all; however, achieving this target requires intensified investment and policy enforcement. For instance, poor WASH infrastructure leads to girls missing school or dropping out altogether, perpetuating educational inequalities and undermining the attainment of SDG 4, which focuses on quality education for all. Moreover, when developing programs for MHH and related WASH facilities, WASH professionals must take into account not only preferences regarding menstrual material selection, technologies, and disposal and washing practices, but also the physical and social perceptions of menstruators, the accessibility and nature of knowledge within the cultural context, as well as the menstrual taboos and social stigma that persistently affect menstruators worldwide (44).

## 3 Conclusion

In conclusion, this paper examined the factors influencing menstrual hygiene knowledge, attitudes, and practices among adolescent girls in rural schools across Africa. The findings highlighted significant gaps in menstrual hygiene knowledge, with many girls demonstrating limited understanding of basic concepts related to menstrual hygiene. These knowledge deficits are often compounded by unreliable or insufficient sources of menstrual hygiene management information and unpreparedness for menarche. The topic is surrounded by prevalent cultural taboos, myths, and stigma surrounding menstruation, which hinder open discussions and education on the topic. This affects their menstrual hygiene practices, which is frequently inadequate, influenced by resource limitations, lack of appropriate guidance, poor access to sanitary products, and insufficient menstrual hygiene education. In particular, the availability of and access to WASH facilities were identified as critical determinants of menstrual hygiene practices. The lack of privacy, clean water, and disposal options in rural schools creates significant barriers to effective menstrual management. Additionally, inadequate infrastructural support within schools exacerbates these challenges, undermining efforts to promote healthy menstrual hygiene practices. These findings highlight the urgent need for comprehensive, multi-sectoral interventions that address menstrual hygiene management holistically. This includes increasing access to menstrual products, improving WASH infrastructure in schools, and implementing culturally sensitive education programs to enhance knowledge and foster positive attitudes toward menstruation among adolescent girls in rural Africa.

Furthermore, there is a notable lack of intervention trials and culturally tailored educational programs that have been rigorously evaluated for their effectiveness in improving menstrual hygiene outcomes. Future research should prioritize designing, implementing, and assessing context-specific interventions that directly address the unique sociocultural and infrastructural challenges faced by adolescent girls in rural African settings. There is also a need for research focusing on policy implementation, cost-effectiveness of free sanitary product distribution, and the integration of menstrual health into school curricula, which also remains limited. Sanitary pad distribution initiatives demonstrate a notable rise in sanitary pad utilisation among young women in LIMC, underscoring the efficacy of these programs in mitigating obstacles such as cost and insufficient information (45).

## 4 Implications

The study highlights the need for governments to prioritize MHM as a public health and education issue, integrating it into national health and education policies. The findings call for the integration of comprehensive menstrual health education into school curricula to dispel myths, reduce stigma, and enhance knowledge among students, teachers, and communities. Educators should be trained to provide accurate, sensitive, and culturally appropriate menstrual health education to ensure effective knowledge transfer. Additionally, policymakers should allocate budgets to provide free or subsidized menstrual products and improve WASH facilities in rural schools.

## 5 Limitations

This review is limited by its inclusion criteria, which focused exclusively on English-language, peer-reviewed, quantitative studies. The exclusion of qualitative and mixed-methods research likely restricted the depth of contextual and experiential insights into menstrual hygiene management. Additionally, publication bias may have skewed the available evidence toward more formal or well-funded studies, while the underrepresentation of francophone African countries limits the geographic comprehensiveness of the findings. Lastly, the review was not initially framed using the Knowledge–Attitude–Practice (KAP) model, which may have constrained the analytic clarity in understanding how knowledge and attitudes translate into hygiene practices (46, 47).
